# Recent advances in nanoparticle-based drug delivery systems for rheumatoid arthritis treatment

**DOI:** 10.1039/d2na00229a

**Published:** 2022-07-26

**Authors:** Simran Nasra, Dhiraj Bhatia, Ashutosh Kumar

**Affiliations:** Biological & Life Sciences, School of Arts & Sciences, Ahmedabad University, Central Campus Navrangpura Ahmedabad Gujarat India ashutosh.kumar@ahduni.edu.in +91796191127; Biological Engineering Discipline, Indian Institute of Technology, IIT Gandhinagar Palaj 382355 Gujarat India

## Abstract

Nanotechnology has increasingly emerged as a promising tool for exploring new approaches, from treating complex conditions to early detection of the onset of multiple disease states. Tailored designer nanoparticles can now more comprehensively interact with their cellular targets and various pathogens due to a similar size range and tunable surface properties. The basic goal of drug delivery is to employ pharmaceuticals only where they are needed, with as few adverse effects and off-target consequences as possible. Rheumatoid arthritis (RA) is a chronic inflammatory illness that leads to progressive loss of bone and cartilage, resulting in acute impairment, decreased life expectancy, and increased death rates. Recent advancements in treatment have significantly slowed the progression of the disease and improved the lives of many RA sufferers. Some patients, on the other hand, attain or maintain illness remission without needing to continue immunosuppressive therapy. Furthermore, a large percentage of patients do not respond to current treatments or acquire tolerance to them. As a result, novel medication options for RA therapy are still needed. Nanocarriers, unlike standard medications, are fabricated to transport drugs directly to the location of joint inflammation, evading systemic and negative effects. As a result, researchers are reconsidering medicines that were previously thought to be too hazardous for systemic delivery. This article gives an overview of contemporary nanotechnology-based tactics for treating rheumatoid arthritis, as well as how the nanotherapeutic regimen could be enhanced in the future.

## Abstract 2

Nanotechnology has increasingly emerged as a promising tool for exploring new approaches, from treating complex conditions to early detection of the onset of multiple disease states.

## Graphical abstract

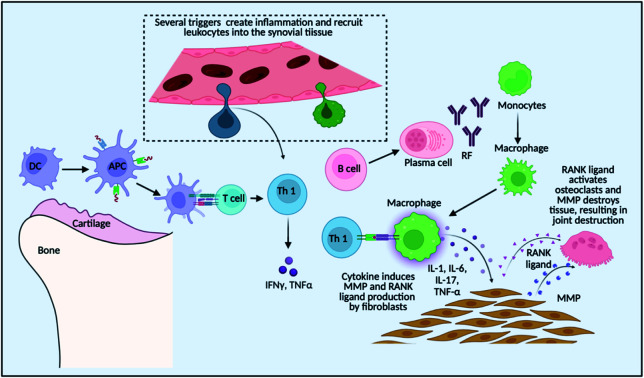

## 1. Introduction

Rheumatoid arthritis (RA) is a chronic autoimmune disease characterised by significant synovial joint inflammation and osteocyte and cartilage destruction. This results in long-term impairment, inability to interact effectively in social life and daily employment, and greater death rates, all of which have a significant influence on the patient's quality of life.^1^ RA is a group of joint diseases characterised by chronic joint inflammation that affects the smaller joints of hands and foot, such as wrists, fingers, and toes.^2^ The following are the main clinicopathological features: inflammatory cells infiltrate the joint, creating synovial inflammation and hyperplasia, a spike in inflammatory factors, and invasion of neighbouring cartilage, resulting in bone erosion and cartilage tissue loss.^3^ Despite significant advances in understanding and experience in the treatment of RA in recent years, effective therapy for RA continues to be a challenge.^4^ Presently, the primary goals of pharmacological therapy are to relieve RA symptoms and reduce disease activity. The European League Against Rheumatism recommends a number of medicines to treat RA.^5^ The guidelines recommend the onset of early treatment with therapeutic drugs such as synthetic and biological disease-modifying anti-inflammatory drugs (DMARDs), which include biological DMARDs like certolizumab pegol, etanercept, and adalimumab as well as traditional synthetic DMARDs like leflunomide, methotrexate (MTX), and also sulfasalazine. Certolizumab pegol encapsulated in nanocarriers with PEGylation augments the time required to reach half of its concentration to fourteen days and it also depicts encouraging outcomes in RA patients.^6^ This emphasises that DMARDs play a critical role in pharmacological approaches to RA treatment that cannot be substituted. Glucocorticoids (GCs) can, however, be utilized as a bridge treatment till traditional synthetic DMARDs show detectable therapeutic properties.^7^

The pharmacological therapy of RA leads to several complications. There are reported adverse effects on the gastrointestinal tract, hepatic, cardiac and renal function because of prolonged usage of DMARDs, NSAIDs (non-steroidal anti-inflammatory drugs) and glucocorticoids. It is necessary to surmount the limitations of these therapeutic agents while curing RA.^8,9,10^ Many nanoparticles have recently been discovered to be capable of delivering RA medicines *via* influencing immune cells such as macrophages in inflamed joints.^11,12^. This method increases the drug solubility and makes it more bioavailable, prevents overdosage and improves patient adherence.^13^ Targeted drug delivery systems are pharmaceutical constructs that help achieve specific delivery of drugs or bioactive molecules to cells or tissues that express a tissue-specific molecular marker that separates them from healthy tissues in the body.^14,15^

**Fig. 1:**
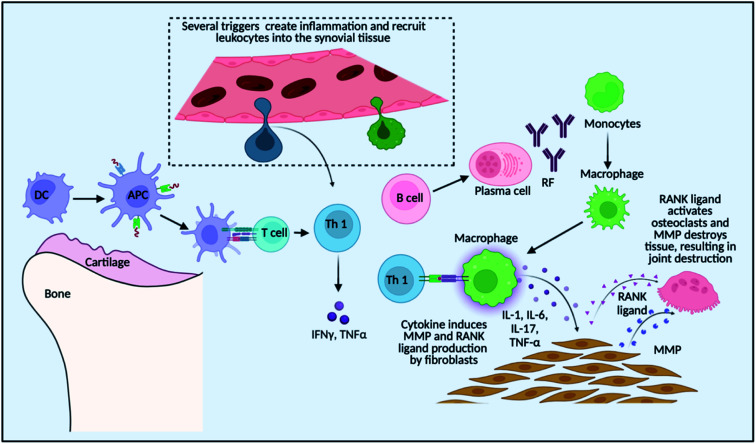
Pathophysiology of RA and role of various immune cells.

These delivery systems penetrate the epithelial barriers and release the drugs, and then these bioactive molecules move across multiple biological barriers to make it to the site of action.^15^ An excellent drug delivery strategy ensures that an effective drug is bioavailable at the desired location.^16^ The drug concentration at the target location should be within the therapeutic window, which is the range between the drug's minimum effective and hazardous concentrations.^17^ Modern drug delivery research and tactics have progressed significantly; yet, many medications have unfavourable side effects as a result of interactions with organs of the body that are not the intended targets for the drug.^18^ As a result of some groundbreaking discoveries, advanced treatments are available for RA patients, for example, methotrexate for RA therapy is significantly effective but is known to have a wide range of side effects if taken for a long duration.^19^ Several cellular receptors and targets have been studied to understand the cellular pathways and molecular targets for targeted therapy, such as using small interfering RNA (siRNA) as a therapeutic substance to downregulate these signalling-specific genes^20,21^. Applied nanotechnology is a promising interdisciplinary research field that combines the expertise of engineers and scientists, resulting in recent advances demonstrating the tremendous potential of nanoparticles in medical applications,^22^ and nanomedicine is rapidly evolving with the ability to target specific and effective drug delivery. It addresses the limitations of conventional therapy, which is confirmed by some preclinical and clinical research showing drug delivery at a specific site, reduced side effects and improved treatment outcomes.^23^ It is an encouraging area for cell-specific and regulated transport of micromolecules and macromolecules in treatment, as it aids in the establishment of stable interactions with ligands, change in the size and shape, ability to combine hydrophilic and hydrophobic materials, and improved carrier capacity.^24^ Nanosystems allow for site-specific and targeted drug delivery while lowering drug consumption, reducing the risk of off-target side effects. In high-risk individuals, these systems may be approved for long-term use of NSAIDs and GCs. Inhibitors of signal transduction pathways that govern inflammation, such as Janus kinase (JAK), splenic tyrosine kinase (Syk), and activated B-cell nuclear factor-k light chain enhancer (NF-kB), have recently been added to the pool of targeted pharmaceutical therapy.^25^ As discussed in Fig. 1, there is a recruitment of various immune cells at the site of synovium inflammation and proinflammatory cytokines released recruit macrophages that further add to the inflammatory milieu.^26^ The anti-rheumatoid effects of nanoparticles for the treatment of RA and schemes to modulate the pro-inflammatory systems are summarised and several research findings that are of clinical application are discussed, in the present review article. Numerous advancements have been made in drug delivery over the last few decades, and there is an ever-increasing need to connect the published studies in order to gain a better understanding and provide a fundamental baseline for future research on fresh concepts.

As shown in Fig. 1, the localized dendritic cells act as antigen-presenting cells (APCs) and activate the T cells that are recruited at the site of inflammation due to inflammatory signals. T cells then further get converted to Th1 (T helper cells 1) that produce pro-inflammatory cytokines like tumour necrosis factor (TNF) and interferon gamma (IFN-γ) that ultimately recruits macrophages which adds to the pro-inflammatory cytokine milieu by secreting TNF and interleukins such as Interleukin-1 (IL-1) and Interleukin-6 (IL-6). These cytokines induce matrix metalloproteinase (MMP) and the receptor activator of the nuclear factor-kB (RANK) ligand in the fibroblast which activates osteoclasts for bone destruction. Also, Th 2 (T helper cells 2) activates B cells to secrete auto-antibodies like rheumatoid factor (RF), which again helps in macrophage recruitment and the inflammation is sustained.

## 2. Limitation of traditional RA therapy and role of nanomedicines in the last decade

**Fig. 2:**
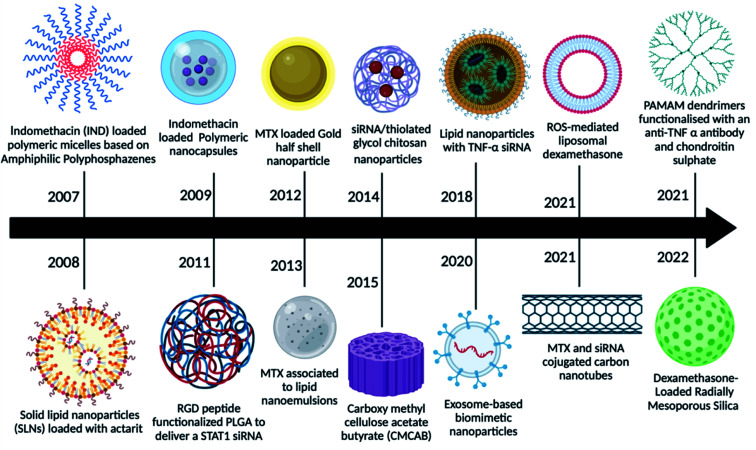
Various nanoparticles used in the last decade for enhanced drug delivery.

The main drawbacks of the traditional medicinal dosage of RA were poor adherence to patients, a rapid half-life, poor bioavailability, and inadequate solubility, all of which might be resolved by looking for novel dosage approaches.^27^ Microparticles, nanoparticles, nanodispersions, nanocapsules, nanoemulsions, nanosuspensions, and other novel delivery approaches for RA therapies boost drug efficacy by delivering the drug at a higher concentration to the target location. As demonstrated in Fig. 2, nanomedicine has grown significantly in use for RA therapy over the last 10–15 years.

In a pilot investigation, indomethacin-loaded polymeric micelles based on amphiphilic polyphosphazene showed promising results, indicating that this type of amphiphilic copolymer can be employed as injectable nano-carriers for hydrophobic medicines.^27^ Later, actarit, a poorly soluble medication, was loaded into solid lipid nanoparticles (SNLs) for intravenous delivery, reducing side effects such as gastrotoxicity and kidney dysfunction. After intravenous treatment in mice, the total targeting efficiency (TEC) of actarit-loaded SLNs increased from 6.31% to 16.29% in the spleen, whereas the renal distribution of actarit was dramatically reduced compared to actarit solution.^28^ Following that, polymeric nanocapsules were employed to deliver indomethacin, which showed enhanced anti-inflammatory potential in long-term treated rat models of inflammation, as well as improved gastrointestinal well-being.^29^ Because RGD is an inflammation-targeting moiety, MTX-loaded poly(dl-lactic-*co*-glycolic acid) (PLGA)-Gold (Au) half-shell nanoparticles (MTX-PLGA-Au) and conjugated arginine-glycine-aspartic acid (RGD) peptides on the surface of the Au half-shell increased the potential of MTX as a RA treatment.^29^ Progressively several other nanomedicines were reported as potential candidates for RA therapy like MTX combined with nanoemulsions,^30^ siRNA-loaded chitosan nanoparticles,^31^ curcumin-loaded carboxy methyl cellulose acetate (CMCAB),^32^ exosomes as biomimetic particles,^33^ stimuli-responsive liposomes,^34^ MTX and siRNA loaded carbon nanotubes,^35^ poly(amidoamine) (PAMAM) dendrimers functionalized with chondroitin sulphate and with anti-TNF-α to provide anti-inflammatory properties^36^ and mesoporous silica nanoparticles encapsulating dexamethasone^37^ to deliver the traditional drugs.

## 3. Transport obstacles faced by nanomedicines

**Fig. 3:**
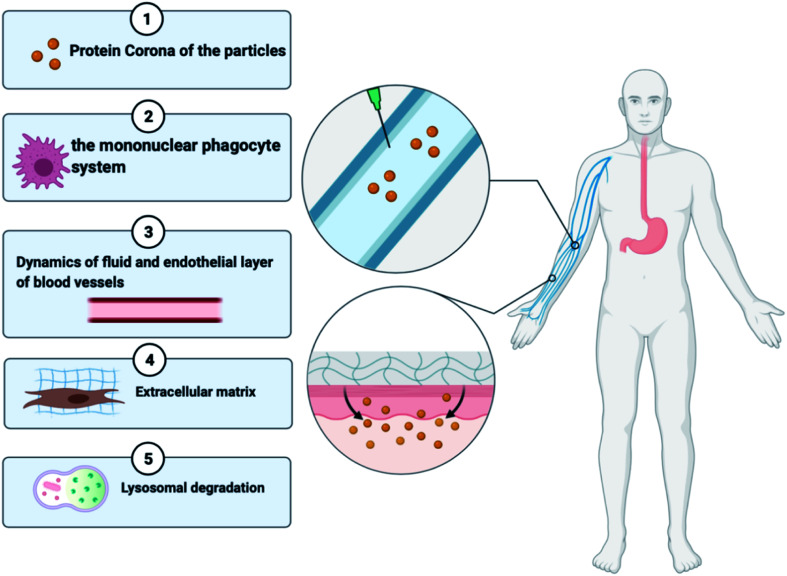
Biological barriers faced by nanomedicines.

Regardless of the benefits presented by nanoparticles, certain types of obstacles faced by the drug delivery systems are stated below in Fig. 3:^38^ (a) the nanoparticles that are not targeted could be identified by the mononuclear phagocyte system available in blood circulation and bone marrow and in organs like the spleen, lung, and liver,^39^ (b) due to the hydrophobicity of nanoparticles, there might be greater adsorption of blood constituents onto the nanoparticle shell, (c) prolonged circulation duration of the nanoparticle is a requirement for *in vivo* injection till it gets to the area of the target, and (d) when it comes to drug targeting, atypical cell assembly can hinder the increased permeability and retention (EPR) effect, resulting in less effective drug internalisation. The protein corona, elimination by the mononuclear phagocyte system (MPS), fluid dynamics, the endothelial surface of blood vessels, the extracellular matrix, the cellular membrane, lysosomal breakdown, and efflux pumps are all biological hurdles that must be surmounted^40,41^,^42^. To overcome these delivery obstacles, the nanoparticles have to be manipulated by functionalizing them with molecular targets. A strategy to overcome the RES is by undertaking an inverse targeting approach. The inverse targeting approach aims to escape the passive uptake of the nanoparticle by Reticulo-Endothelial Systems (RESs) and therefore the method is denoted as inverse targeting. RES standard function is repressed by introducing a substantial quantity of blank delivery systems or any other moieties like dextran sulphate which subsequently directs to the overload of the RES and suppression of immune response.^43^ All the colloidal transport systems like micelles, fluid crystals, vesicles, and dispersals of nanoparticles containing smaller molecules exhibit the potential for successful outcomes for target-specific approaches for drug transport. The target is to improve the overlay of the drug and release kinetics and prolong the shelf lifespan of the drug with fewer side effects.^44^ A drug with the colloidal mixture displays liquid and crystal structures or amphiphilic features which may alter the molecular interactions of the drug in the biological system. Covering of hydrophobic particles with hydrophilic materials decreases the uptake by the RES, thereby permitting the carriers to reach other various sites.^45^

As shown in Fig. 3, a broad category of processes of transport involves transportation within distinct compartments like in cytoplasm and among separate compartments. Various biological barriers like cell, nuclear, and endosomal membranes have a major interference on drug delivery. (1) Nanoparticles collect molecules on their surface when in contact with the biological fluid developing the “protein corona”, which can affect their future therapeutic execution. This biomolecular overlay gives a particular identity to a nanoparticle, determining how they are seen by and interact with cells and contributing to their internalization and distribution. (2) The activated macrophages of the reticuloendothelial system help in the circulatory removal of old blood cells and blood-borne substances into the RES organs. A key limitation of the nanotherapeutic formulation is its quick phagocytosis and RES-based clearance, which reduces the bioavailability of the particle. (3) The nanoparticles face a lot of complex fluid forces in the bloodstream as they shift through the curvatures and bifurcates of healthy or unhealthy blood vessels^46^. (4) The extracellular matrix imparts structural integrity to the tissues and contains a higher collagen content which provides rigidity and serves as a barrier for the transportation of nanoparticles. (5) Once the particles are extravasated from the blood vessels to the location of the infection, they must bind to the cell membrane resulting in the endocytosis of the particle. The nanotherapeutic agents are then localized in late endosomes and the nucleus, degraded in lysosomes or recycled back to the plasma membrane or escape this lysosomal degradation and any other organelles. This suggests that the nanoparticles face a range of obstacles when administered intravenously or intradermally; however, if there is a development of nanomedicine that targets a molecular moiety or any immune cell involved in the pathophysiology of RA and administered at the site of inflammation then it can surpass most of the transport obstacles. Various studies show enhanced passive and active targeting of drugs by modifying the surface of nanoparticles as discussed in Sections 4.1 and 4.2.

## 4. Approaches to target inflammatory synovial macrophages

According to the reported literature, the mechanism of tailored nanoparticles to transport medicines in the treatment of RA is still mostly unknown.^47^ It was known that macrophages are more common in inflamed joints and that nanocarriers may be successfully absorbed and phagocytosed by macrophages with no surface modifications; therefore targeting immune cells like macrophages using a nanocarrier system was a way that was examined on priority.^48^ To control macrophage-mediated production of pro-inflammatory cytokines, some of the early research used a low dose of clodronate, a bisphosphonate that triggers apoptosis in phagocytic cells and is contained in liposomes (unilamellar or multilamellar lipid vesicles with a mean size of 100 nm).^49^ Rabbits receiving weekly intra-articular injections of low-dose liposomal clodronate saw a transitory reduction and delay in joint swelling, but the effect did not extend beyond the first week.^50^ Because greater doses of liposomal clodronate generated synovitis and pro-inflammatory effects, it is doubtful that this technique will be used in the treatment of arthritis.^51^ Parenteral delivery of nanoparticle systems has been successfully used to target macrophages.^52^ These methods take advantage of the fact that macrophages play a key role in the progression of RA (by producing pro-inflammatory cytokines) and that nanoparticles can be consumed by macrophages *via* leaky inflamed capillaries into arthritic joints, a phenomenon known as increased permeability and retention (EPR). However, systemically injected nanoparticles can be quickly eliminated by macrophages in the reticuloendothelial system (RES), reducing the number of drug moieties available at the location of RA-affected joints.^53^ As a result, modifying nanoparticles to delay RES interaction and selectively target different organ systems, immune cells, or specific pathways is constantly being investigated.

### 4.1. Nanoparticles for passively targeting macrophages

Increased vascular permeability and macrophage infiltration are two pathologic features of RA, both of which might provide favourable circumstances and target cells for nanomedicine delivery systems. The ELVIS effect (Extravasation *via* Leaky Vasculature and Inflammatory Cell-mediated Sequestration) allows the nano-drug carrier to preferentially concentrate and release medications in synovial tissue, comparable to the improved permeability and retention effect reported in the therapy of tumours.^54^ Because nano-drug particles are bigger than 200 nm and smaller than 10 nm, they can be removed by the spleen and renal tract. Hence, the particle size is an important factor to consider when developing a passive targeting method. As a consequence, only nanoparticles conjugated with drugs with a size range of 100 to 200 nm were able to escape from being removed by the mononuclear phagocyte system and the reticuloendothelial system (RES) and stay in blood circulation for an extended period of time in RA patients.^55^ The following are some nanoparticle modifications for better passive delivery of drugs and fewer off-target effects.

#### 4.1.1. Surface modification of nanoparticles with PEG

**Fig. 4:**
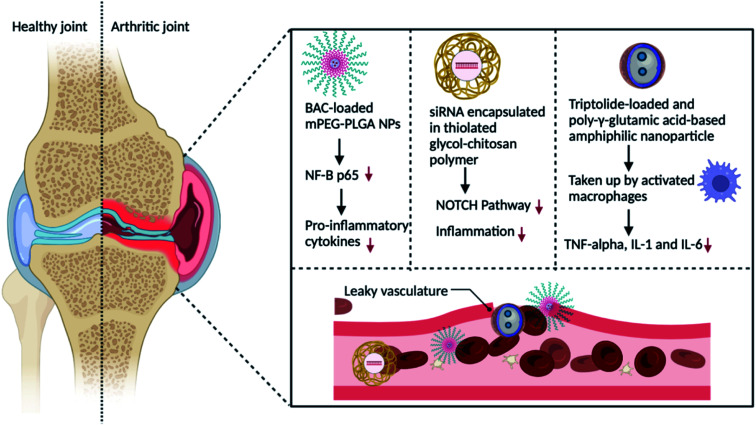
Schematic representation of the passive drug targeting delivery strategy for the treatment of RA by altering macrophages with nanoparticles containing a therapeutic drug.

PEG is a hydrophilic polymer that stands for polyethylene glycol. PEGylated nanocarriers are shown to collect more in inflammatory synovium and are removed to a lower level in the spleen and liver.^56^ As discussed in the reported work of Heo *et al.,*^57^ researchers employed dexamethasone palmitate (DXP), a hydrophobic prodrug of dexamethasone (Dex), and DSPE-PEG2000 to generate PEGylated DXP NPs. DXP nanoparticles were created using the hydrophobic interaction between the stearic acid chain of PEG lipids and the palmitic acid sequence of dexamethasone palmitate. The nanoparticles were shown to be extremely efficient in preventing DXP crystallisation and avoiding poor-drug loading capacity and progressive suspension instability. Furthermore, the PEG on the nanocarrier's shells can form a system that is indistinguishable from the MPS and RES, allowing for long-term blood circulation.^58^ The ATCM monomer benzoylaconite (BAC) has been synthesised in PEG-modified nanomedicine to modulate macrophages in the treatment of RA. Scientists generated a methoxy-PEG-poly(lactide-*co*-glycolide) copolymer known as mPEG-PLGA by ring-opening polymerization, which they dissolved in DMSO and BAC in methanol. Finally, the mPEG-PLGA and BAC suspensions were combined to form NP/BAC, BAC-loaded mPEG-PLGA NPs.^58^ The nanoparticles gathered in RA joints and released BAC, which reduced NF-B p65 overexpression, inhibited the generation of pro-inflammatory cytokines, and reduced inflammation (as shown in Fig. 4).

Small interfering RNA (siRNA)-assisted gene silencing has also been employed in the treatment of RA.^59^ Chemical modification is necessary to alleviate these shortcomings due to its lower stability and permeability. Another significant macrophage kinase that can induce pro-inflammatory macrophage polarisation is Bruton's tyrosine kinase (BTK). Furthermore, reducing BTK expression in these macrophages can lower inflammatory cytokines, halt RA development, and decrease the RA symptoms.^60^

#### 4.1.2. Modifications of nanoparticles with chitosan

Chitosan is a natural-based polysaccharide loaded with amino chemical moieties that are extensively exploited in biomedicine because it is biocompatible and exhibits bioactivity. It is also non-toxic and biodegradable. The study of chitosan by-product's amphiphilic characteristics has recently piqued interest.^61^ Chitosan amphiphilic copolymers, such as glycol chitosan, which is produced *via* hydrophobic alteration of chitosan, could be circulated in an aqueous suspension and have a long circulation time. In a study, siRNA aimed to target the Notch pathway was encapsulated in a thiolated glycol-chitosan polymer that achieves extended cycle physical characteristics *in vivo* and freely assembles in inflamed joints (as indicated in Fig. 4).^62^

#### 4.1.3. Biomimetic nanocarriers

According to the current research, PEGylated polymer nanoparticles induce a strong immune response that can boost blood clearance.^63^ As a result, a biomimetic drug delivery method based on natural nanoparticles was developed, which camouflaged nanoparticles as autologous elements to degrade their ability to elicit a robust immune response, evade immune defence, increase the circulation duration, augment aggregation at the target site, expand therapeutic efficacy, and reduce toxic and off-target effects.^64^

l-aspartic acid is a necessary amino acid comprising intriguing features for functionalizing iron oxide nanoparticles. It is also affordable and biocompatible. It is widely utilised in drug and chemical synthesis, and it has a substantial medical application. The high zeta potential, hydrodynamic size, and low polydispersity index of these nanoparticles, together with their non-toxic effect, considerably boost the possibility of using them in *in vitro* and in *in vivo* biomedical sectors such as cell labelling, medication and diagnostic delivery therapies. This research lays the framework for the efficient synthesis of aspartic acid(AA)-iron oxide nanoparticles (IONPs) for future *in vitro* and *in vivo* studies (pending) of AAs-coated IONP conjugates with medicines, drug candidates, and molecular probes.^65^ Triptolide (TPT), a TCM single unit, was coated on poly-glutamic acid-grafted di-*tert*-butyl-l-aspartate hydrochloride, which was formed by the amidation chemical reaction between aspartic acid and poly-glutamic acid in one study. Nanoparticles accumulating in RA joints *via* the ELVIS effect were picked up by activated macrophages and free TPT, which reduced pro-inflammatory cytokines such as TNF, IL-6, and IL-1.^66^ Nanocarriers could be used as endogenous vesicles that combine with biological cellular plasma membranes, reducing mononuclear phagocyte system elimination and boosting circulation time. They activate macrophages by recognising CD44 (cluster of differentiation 44) or macrophage antigen 1 (Mac-1), stick to the endothelium to target the inflammatory region of RA for drug administration, and depict macrophage mimics to cooperate with NF-kB and colony-stimulating factor (CSF) receptor activator (RANKL) for osteoclast prevention^67,68^.

As shown in Fig. 4, when administered intravenously, nanocarriers aggregate in the RA joints because of the ELVIS effect. Consequently, the nanocarriers are taken up by stimulated macrophages and specifically transport the therapeutic agents like celastrol, benzoylaconitine, methotrexate and Notch1 siRNA *via* pH, ROS and redox-responsive methods. This procedure diminishes the secretion of TNF, MCP-1 (monocyte chemoattractant protein-1), IL-6, and IL-1, thus decelerating the advancement of RA.

### 4.2 Nanoparticles for actively targeting macrophages

Active drug delivery represents particular communications between the therapeutic agents and nano-carrier with the cells of the target, frequently *via* a certain ligand-receptor communication.^69^ The interactions between the ligand and receptor are feasible when these constituents are in direct contact with each other.^70^ The carrier system transporting the drug moves to a targeted location with the help of surface modification that was functionalized on the surface and not get biologically taken up by the RES. The alteration method involves a coating of the surface with a biological bonding agent, non-ionic surfactant and specific cell or antibodies or by albumin protein. Particular interactions of the ligands on the external area of nanoparticles and specific receptors present on the target cells have enabled the intake of the complex of the receptor and nano-carrier by receptor-based endocytosis and vesicle-based transportation *via* endosomes.^71^ Active targeting approaches have been established to surmount the limitations and improve the accumulation of nanocarriers at the inflamed joint. The main procedure of active targeting is attained *via* surface alterations of nano-drugs with location-targeting ligand molecules. RA-affected cells exhibit an elevation in the membrane receptors or surface protein levels on their exterior that improves the proficient internalization of nano-carriers *via* receptor-aided engulfment, which aids the nanoparticles to enter inside the cells. The drug-loaded nano-carriers can arrive in the affected cells *via* such ligand and receptor-mediated interaction. After reaching the targeted tissue area, the nano-carriers may employ cytosolic action after the intake by the cell or at the level of the cyto-membrane.^72^ The release of the drug from its vehicle may occur on the surface of the cell, in the extracellular matrix, or through the lytic and digestive enzymes existing in the lysosomal organelles. This ensures the discharge of the drug into the cytosol without the attachment of the colloidal carrier moiety.^73^ The proteins or the receptors must be salvaged back to the exterior membrane of cells. Such a mode of targeted drug delivery comprises of some vital components like nanoparticles onto which the targeted drug can be attached, like ligands or antibodies (IgG) are functionalised on the surface and it augments the target of nanoparticles towards that specifically targeted antigenic proteins or the cell membrane receptors.^74^ Scientists used small ligands such as folate, class A scavenger receptors (SR-As), and galactose for nano-drug modification to target different specific receptors overly expressed on the exterior of macrophages, such as folate receptors, class A scavenger receptors (SR-As), and galactose receptors, which then targeted macrophages.^75^

Different targets are linked with a considerable number of factors engaged in synovial fluid inflammation and they have been investigated for RA therapy. One such target is the folate receptor, which is expressed highly in macrophages residing in the synovium of RA affected joints.^76^ The chemical conjugation of folate to different molecules by the carboxyl group permits the formation of folate conjugates which are taken inside by receptor aided endocytosis. Many studies have recognized the CD44 receptor expressed in a high amount in the synovial macrophages, lymphocytes, and fibroblasts of RA-affected patients as a potential target by linking with its ligand molecules like hyaluronic acid. CD44 is a type of adhesion related receptor broadly allocated in epithelial cells and stimulated lymphocytes.^77^ CD44 expression was upregulated in the macrophages present in inflamed joints and fibroblasts. Hyaluronic acid is a polysaccharide actively utilized in delivery systems for drugs or in engineering of tissues as a ligand molecule for the CD44 receptor.^78^ Some studies have prepared hyaluronic acid linked lipid nanoparticles with prednisolone (PD) by covering GC-PD in hyaluronic acid-coated small lipid nanoparticles. This showcases increased stability and drug loading efficiency, as well as the ability to safeguard the unstable therapeutic drugs from degradation.

A PEGylated liposome targeting macrophages was created by Duan *et al.* The anti-RA FDA authorised medication, methotrexate, was encapsulated into the lipid bilayer of liposomes, while siRNA encapsulated in calcium phosphate nanoparticles was packed in the polar liposomal core. The transmembrane transport capacity and drug delivery features of these drug-loaded nanocarriers were improved. For years, MTX has been used to treat RA as a single agent or together with other medications. Long-term usage of MTX, on the other hand, causes side effects and drug resistance. As a result, combining siRNA with MTX for RA treatment could have a synergistic impact. Because siRNA could not be stable on its own, numerous ways of siRNA transport, such as polyethyleneimine (PEI) and dendrimers, have been devised. The ability of such particles to preserve siRNA and mediate cellular uptake into target cells has been demonstrated; however, a strong positive charge is detrimental to siRNA activity. As a result, the authors developed calcium phosphate nanoparticles (CaP) for siRNA delivery in this work. They built a PEGylated liposome that can load siRNA/CaP in its core while MTX is loaded in the lipid envelope of the liposomes to boost the stability of the siRNA/CaP complex and load the MTX. Additionally, the PEGylated liposome was later functionalized with folic acid (FA) and could only target active macrophages that overexpressed folate receptors. This resulted in the expression of NF-kB p65, which is implicated in the inflammatory signalling cascade, being silenced, lowering inflammatory cytokines, and encouraging the conversion of M_1_ macrophages to M_2_ macrophages for RA therapy. Combinatorial nanoparticles were more successful than individual medications at blocking NF-kB signalling pathways and reducing the expression of pro-inflammatory cytokines.^79^ Nanoparticles modified with PEG agglomerate widely in RA joints, enter the cell membrane *via* Sta-R8 (stearic acid-octa-arginine), and are absorbed by active macrophages *via* folate receptor-aided endocytosis. Polyketal (PK) also acts as a pH-sensitive switch, dissolving in an acidic environment and releasing methotrexate to prevent the release of pro-inflammatory cytokines. Folic acid-modified silver nanoparticles are also being investigated in order to target M_1_ macrophages and drive the release of Ag^+^ ions due to glutathione's action. This caused M_1_ macrophages to go into apoptosis and reduced reactive oxygen species, allowing them to switch from M_1_ to M_2_, reducing inflammation and improving efficacy.^80^ Similarly, liposome loaded prednisolone with surface modification of PEG has been under clinical trial phase II since 2016; however, no progress has been made ever since (NCT02495662).

#### 4.2.1. Liposomes in actively targeting synovial macrophages

**Fig. 5:**
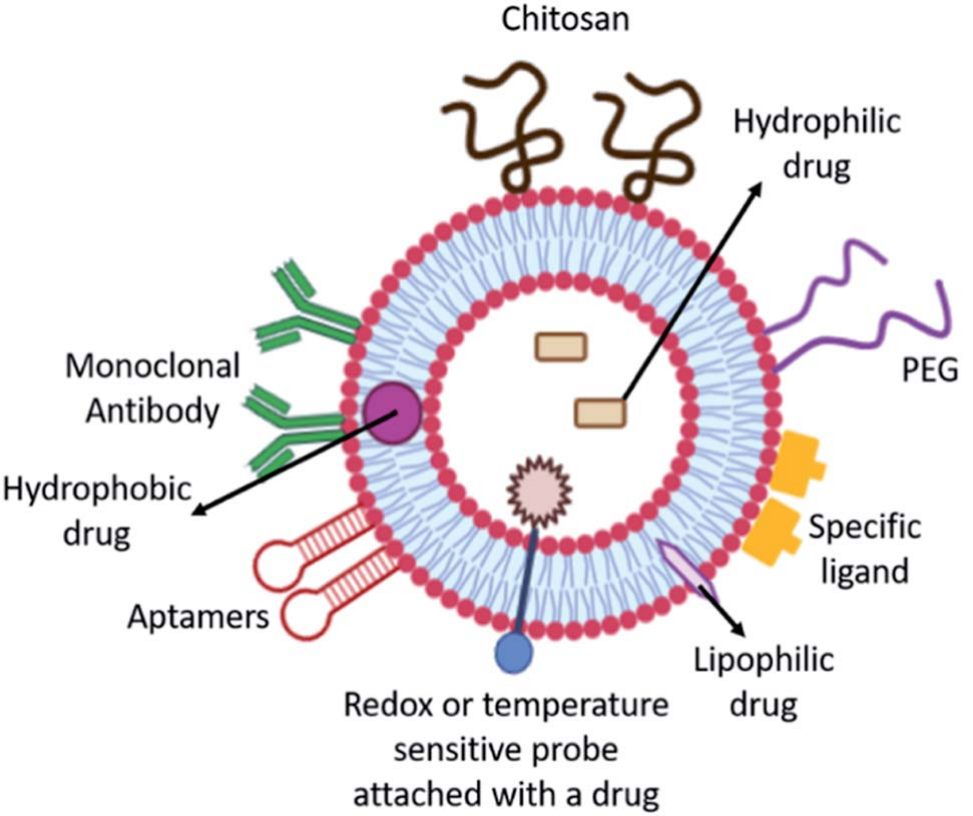
Schematic of various strategies to utilize the hydrophobic bilipid layer and hydrophilic core of liposomes for efficient delivery of RA therapies.

Liposomes are sphere-shaped nanocarriers comprising a bilayer membrane of phospholipids as the exterior surface and a polar core.^81^ Liposome's chemical and physical properties make them a good delivery vehicle since therapeutic agents can be enclosed in the polar cavity or between the bilipid outer layers. For the treatment of rheumatoid arthritis, liposomes have been widely used. PEG is a hydrophilic polymer that can reduce liposome recognition and uptake by the RES, resulting in prolonged liposome circulation in the body.^82^ To boost the therapeutic efficacy of dexamethasone in RA, stealth polymeric liposomes can be used as nano-carriers. In arthritic animals, liposomes can preferentially collect in inflamed joints, inhibiting high levels of pro-inflammatory characteristics in RA joints and reducing inflammation and joint enlargement. This shows that polymer stealth liposomes can be used as a new medication delivery technology for a variety of medicinal purposes.^2^ iRGD-peptide-functionalized echogenic liposomes (iELPs) were also exploited to load methotrexate comprising indocyanine green fluorescent probes for tracing fluorescence, and the release of drugs was triggered by ultrasound having a lower frequency. iELPs combine with the v3 integrin in the synovial joint, significantly improving the efficacy of the drug. The infiltration of immune cells and formation of newer blood vessels in joint tissue were considerably decreased compared to untreated RA mice^83^ (Fig. 5).

#### 4.2.2. Polymer-based nanocarriers used in actively targeting synovial macrophages

Polymer nanocarriers are biocompatible, can target specific tissues, and are extensively used for drug delivery. The therapeutic agents for RA can be linked to the surface or loaded in polymer nanoparticles to deliver it to RA joints. The most commonly used method is the surface alteration of nanoparticles with PEG.^84^ This approach can augment the stability of nano-drugs, and it can also reduce immunogenicity and escape from elimination from the body. Polycaprolactone-polyethylene glycol-polycaprolactone (PCLPEG) micelles are aimed at delivering a low dosage of dexamethasone to treat arthritis. The micelles survived in the systemic circulation for a long time and were extensively aggregated at the location of inflamed joints. The delivery of dexamethasone *via* micelles could efficiently decrease the swelling, bone destruction and inflammatory factor expression in blood and joints. The adverse effects of PCLPEG micelles on body mass, T-cell and B-cell counts and glucose concentration in blood, were significantly lower compared to bare dexamethasone, and the complement system activating ability is comparatively weaker.^85^

An alginate nanoparticle comprising IL-10 plasmid DNA and the surface of the nanocarrier are modified with Tuftsin peptide to target macrophages. Tuftsin-modified alginate nanoparticles were aggregated in the inflamed paws of arthritic rodents as observed with the help of near-infrared imaging.^86^ MRI and histological assays exhibited that the nanoparticles significantly decreased the expression of pro-inflammatory cytokines (TNF-, IL-1 and IL-6) in joint tissue and prohibited the progression of inflammation and joint destruction. Following the treatment with nanoparticles, macrophages polarised from M_1_ to M_2_. This research looked into a new way to treat RA. For poly(caprolactone)–poly(ethylene glycol)–poly(caprolactone) (also known as PCLPEGPCL), a ring-opening copolymerization procedure was used to make a triblock copolymer that was used to make methotrexate-loaded nano-cells.^87^ Nanocarriers are also placed in a hydrogel, with eucalyptus oil functioning as a penetrant for dermal administration, which can improve patient compliance while lowering hepatic toxicity. Nanoparticles administered to RA mice can aggregate in inflamed joints more effectively than plain methotrexate, lower inflammatory factor expression levels, and restore the mice's behavioural response capabilities.^88^

#### 4.2.3. Metallic nanoparticles used in actively targeting synovial macrophages

Metal nanoparticles could be utilized by altering it with various functional groups and are utilized in biomedical applications. Currently, Au, Fe, and cerium nanoparticles are frequently utilized for the treatment of RA.^89^ RGD-linked Au half-shell nanocarriers comprising methotrexate are synthesized for the specialized chemo-photo-thermal treatment of RA and these peptides can target sites of inflammation.^90^ Under NIR (near-infrared) radiation, the Au half-shell produces heat to increase the rate at which the drug is released, meanwhile transferring heat and the drug to the inflamed joint. In combination with NIR, nanocarriers comprising a low dosage of methotrexate exhibit better improvement in collagen-induced arthritic mice than methotrexate used alone. The complex of hyaluronate-gold nanoparticles/tocilizumab has been investigated in collagen-induced arthritic mice. Tioconazole is an IL-6 receptor inhibitor that can disrupt the function of IL-6 in the progression of RA, and hyaluronic acid shields cartilage and promotes joint lubrication. The HA-Au-NP/-TCZ complex was studied using ELISA, histological exams, and western blot to determine protein concentration. The results obtained were substantial.^91^ The use of manganese ferrite/ceria co-modified mesoporous silica nanocarriers (MFCMSNs) to treat RA is also being investigated. Hypoxia and inflammation could be reduced by injecting these MFCMSNs. Nanoparticles can down-regulate M_1_ macrophages and drive M_2_ macrophage transition by scavenging ROS and producing oxygen. Silica nanospheres have the potential to release methotrexate in a long-term manner, improving therapeutic efficacy.^92^

## 5. Effects of nanoparticle targeting synovial macrophages in RA

**Fig. 6:**
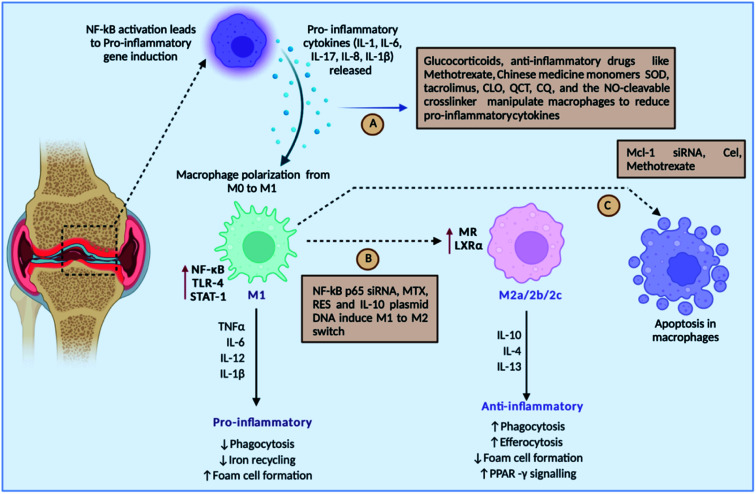
Roles of different drugs either targeting pro-inflammatory cytokines, or inducing macrophage polarisation or inducing the apoptosis of inflammatory macrophages.

The mutual regulation of different cytokines forms a complicated network in the pathogenesis of RA. Cytokines are tiny molecular proteins that operate as intercellular communication mediators. Throughout the inflammatory process, they play a critical role in responding to numerous stimuli. The development of RA is thought to be caused by an imbalance of pro-inflammatory and anti-inflammatory cytokines. Overproduction of pro-inflammatory cytokines, as well as a lack of anti-inflammatory cytokines, can easily result in RA^93^ (Fig. 6).

Fig. 6 discusses the strategies undertaken to modulate macrophages: (A) drug-loaded nanoparticles can target pro-inflammatory cytokines such IL-1, IL-6, IL-8, IL-1, and IL-17 released by macrophages for their activation, (B) some strategies target switching of the M_1_ macrophages to the M_2_ type, which is anti-inflammatory in nature, and (C) meanwhile several drugs leads to apoptosis of inflammatory M_1_ macrophages, such as SOD-superoxide dismutase, QCT-quercetin, CQ-chloroquine, NO-nitric oxide, NF-kB-nuclear factor-kappa B, siRNA-small inhibitory RNA, RES-resveratrol, IL-10-interleukin-10, Mcl-1-myeloid cell leukaemia-1, MTX-methotrexate, and Cel-Celastrol.

The basic causes of RA are pro-inflammatory cytokines. As a result, targeting pro-inflammatory cytokines has been suggested as the main treatment strategy for RA. Also known as M_1_ macrophages, activated macrophages boost inflammation and speed up the clearance of intracellular infections, and in RA, M_1_ macrophages are overproduced, causing increased inflammation and bone damage.^94^ Repolarising M_1_ macrophages into M_2_ macrophages has gained awareness in the therapy of RA. Downregulation of apoptosis can lead to a larger number of macrophages in RA. Hence, targeted inhibition of macrophages and activating apoptosis can augment the treatment regimen.^95^

## 6. Microenvironment-specific nanoparticles

Therapeutic agents that modify the interstitial space or the stromal environment can expand the drug infiltration. A less common strategy to improve drug delivery involves the use of priming agents that modulate the host environment in ways that support drug accumulation specific to the target area. To obtain targeted drug delivery by priming methods to avoid the drug uptake by healthy cells, the following strategies can be undertaken: suppression of resident macrophages in the liver and spleen reduces nanoparticle uptake in healthy organs, inhibition of the complement system may reduce nanoparticle recognition and clearance by the mononuclear phagocyte system, minor hypothermia augments vascular permeability, inhibition of fibroblasts diminishes the production of extracellular matrix elements, and target cell apoptosis expands the interstitial space, leading to improved target distribution of nanoparticles.^96^ There are various stimuli-sensitive nanoparticles that release the therapeutic agents at the site of inflamed joints in response to the disease-modified microenvironment.

### 6.1 Stimuli-responsive nanoparticles and dual delivery system

Target-specific delivery approaches are stimulated by various stimulants such as thermal regulations, pH, and oxidation–reduction potential and a few ailments have dual stimulants nearby the preferred surroundings at the same moment. Smart drug transport approaches are potential candidates for temperature-sensitive and pH-reactive stimulated areas of drug transport.^97^ Several bifurcated polymers hold the capability of combining binary stimulation that have been formulated exhibiting rational applications in various diseases.^98^ Various studies have concluded the sustained and maintained release of drug delivery systems by enzyme-degradable polymers sensitive to varying pH, temperature responsive hydrogels, dual receptive micelles for varying redox environments, and high-intensity focused ultrasound. Hydrolases like proteases, lipases and glycosidases, and oxidoreductases are utilized in the enzyme link to overcome the challenges faced because of variations in pH and enzymatic reactions in between each tissue which can essentially be exploited and serve as triggers for gradually releasing the active drug in the site of action. This would increase the cellular internalization of the nanoparticles at different penetration depths of NPs to release the drug at the site of action. Flurbiprofen encapsulated polymeric nanoparticles responded to a change in pH levels at the site of inflammation. Chitosan glycerin borax was used to formulate thermo-sensitive hydrogels to encapsulate dexamethasone that decreased the pain and progression of inflammation in mice models. Polyethylene glycol−phenylboric acid−triglycerol monostearate was used as a dual stimuli vehicle for targeting dexamethasone at the inflammation area, by pH stimuli and elevated levels of MMPs.^4,99^.

In reaction to diverse stimuli from the micro- or macro-environment, stimulus-sensitive polymers change their physical properties abruptly.^100^ Changes in the form, physical state, solubility, hydrophilic/lipophilic balance, solvent interactions, and conductivity are all responsible for important polymer transitions. Simple chemical reactions such as reduction, oxidation, acid–base interaction, or hydrolysis of units linked to the polymer chain are the driving forces underlying these modifications. The polymers can return to their original state if the trigger is withdrawn, and therefore some of the changes are reversible. External stimuli can trigger irreversible link rupture, resulting in degradation and a significant conformational shift in the polymer structure.^101^ Polymeric nanoparticles (PNPs) make up a large proportion of stimuli based nanoparticles, like nanocapsules, nanospheres, liposomes, micelles, dendrimers, *etc.*

**Fig. 7:**
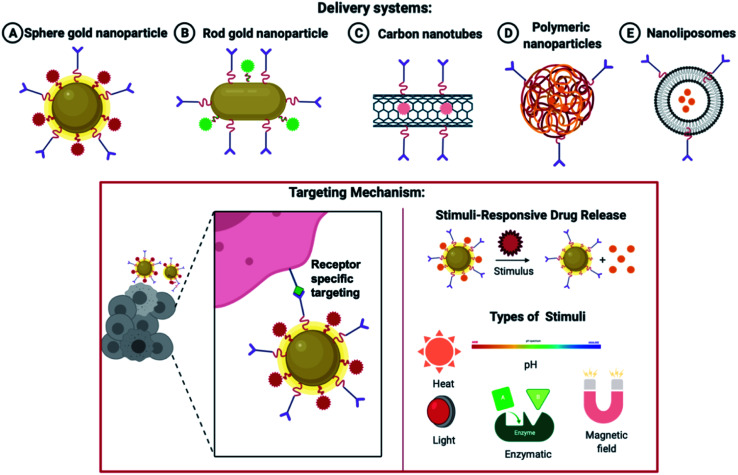
Various nanoparticles are used in drug delivery systems and the release of drugs based on a specific stimulus.

Various delivery systems ranging from gold nanoparticles to polymeric and liposomal-based delivery systems are widely accepted. As shown in Fig. 7, the nanoparticles are decorated with a targeting ligand and directed to a specific RA marker. On reaching the site of action, the nanoparticles can be made sensitive to a particular stimulus like pH, temperature, heat, enzymatic degradation, ultraviolet light, *etc*, which mediates a site-specific and more profound delivery of nanocarriers.

Systemic side-effects may arise if the premature and rapid release of the drug takes place from the nanoparticles. Similarly, a very relaxed release of the therapeutic molecule may shrink the effectiveness of the medicine, elevate the toxic effects due to prolonged exposure and lead to multiple drug resistance. This strategy helps in enhancing the effectiveness and the bio-accessibility of the drug. This area is keenly investigated to attain target delivery and regulated and monitored release of the encapsulated drug. Both endogenous and exogenous stimuli can be utilized. Some endogenous stimuli are pH varying-sensitive,^102^ ROS (reactive oxygen species) level-sensitive,^103,104,105^ environmental redox level-sensitive, responsive to a particular enzyme^106^ and thermo-sensitive delivery strategies^107^ pertaining to a few disease locations. Widely utilized exogenous stimuli contain photo-triggered^108,109,110^ and thermo-triggered strategies.^111^ Magnetic field-activated^112,113^ and X-ray generated^114,115^ drug delivery schemes are also utilized in the device of such trigger-responsive structures. Different stimuli-responsive techniques holding sensitive fragments can be utilized for transferring therapeutic agents to a specific tissue and attaining a drug release as and when required. The characteristics and structures of the trigger-responsive nanoparticles can be altered *via* endogenous or exogenous triggers for enhanced intake by the cellular compartments and the permeation efficacy (as discussed in Fig. 7).^116^

For the monitored release of the drug at the area of pain triggered by ultrasound, a particular sonosensitizer can be triggered to generate ROS. It acts as a remote-controlled release inside the body. Such ultrasound triggers exploit the idea of using localised heat at the site of delivery which in turn discharges the cargo as the delivery system is disrupted.^111^ The product generated after the ultrasonic trigger interacts with the membrane of the liposome and releases drugs due to the peroxidation of unsaturated lipids present in the membrane.^117^ Such a strategy gives flexibility to regulate the concentration and extent of local anaesthesia release, also no other tissue injury was detected.^118^ It was suggested that the sonosensitizer discharges ROS when an ultrasonic trigger was given to the liposome. The elevated levels of ROS then subsequently peroxidates the unsaturated membrane lipids, which ultimately leads to the release of the pre-determined anaesthetic. An external trigger like the magnetic field, luminescence, heat, and sound is of minor influence, inconvenient and realistically not very practical compared to internal triggers like temperature, pH, redox potential, and an enzyme. The encapsulation of medicinal molecules in nanoparticles formed of pH-sensitive building blocks is required in the traditional design of stimuli-based nanocarriers. In an acidic environment, however, drug delivery systems have a tendency to release drugs suddenly rather than gradually.^119^

Furthermore, the delivery techniques face a limitation of appropriate medication loading capacity. Poor compatibility between enclosed cargo and nanocarriers could be the source of these events.^47^ As a result, it's critical to start looking for new ways to improve the design of stimuli-dependent nanoparticles. Ketals were used to make a dexamethasone prodrug with variously structured moieties. These prodrugs are laden onto DSPE-mPEG2000 to devise nanoparticles for RA therapy. Improved interaction between dexamethasone and DSPE-mPEG2000 was observed and better stability with an augmented encapsulation percentage was attained by altering the drug with stearyl alcohol and other long-chain fatty acids like 2-nonadecanol.^120^

The photo-sensitive drug delivery scheme is dependent on the light-absorbing qualities of the nanoparticles, that face a transition of phase after irradiation of light. A response is generated mainly to UV and NIR light. These nanoparticles can control the spatio-temporal activation during the drug release, along with water solubility. Two primary approaches can be used, (a) release of all the drugs together or (b) release at different time points,^121^ because of the tendency of some nanoparticles to exhibit modifications in the structure due to light stimulation, and some nanoparticles can reverse back to the original state like azobenzene derivatives.^122^ Redox-dependent nanoparticles are a developing area for the formulation of novel drug delivery systems.^123^ Arias *et al.* created magnetically sensitive iron/ethylcellulose nanocarriers encapsulated with diclofenac sodium for the therapy of RA.^124^

## 7. Conclusions

Although targeted therapy is commonly regarded from a molecular view, specific cell populations can be targeted through the alteration of drug transport in the body. In addition to the design of nanocarriers, priming strategies represent a promising approach for improving drug delivery. Despite the fact that targeted medications have been beneficial in treating RA, different targeted carriers have a number of drawbacks, including a poor safety profile. Toxicity might result from the spread of carriers to non-target tissues. Surface PEGylation of nano-medicines can help them avoid being recognised by the RES, extending their bioavailability in the bloodstream and increasing their aggregation at the site of inflammation. This promotes the nanocarriers to penetrate and absorb the tissues. The majority of the procedures utilised to make nanoparticles for RA treatment are complicated, with high clinical expenses. Despite the fact that exogenous biomaterials are not hazardous to humans, they can trigger an immunological response. Modern research can target these flaws in RA medication delivery methods and attempt to overcome them. This review study focused on the gains achieved in research on nanocarriers in RA treatment, as well as the drawbacks of present pharmaceuticals and the benefits of nanomedicines, as well as the methodologies and implications of altering immune cells with nanoparticles to better guide future findings.

## Conflicts of interest

There are no conflicts to declare.

## Supplementary Material
